# Evaluation of Full Thickness Wounds Following Application of a Visco-Liquid Hemostat in a Swine Model

**DOI:** 10.3390/pathophysiology31030034

**Published:** 2024-08-29

**Authors:** Michelle Tucci, Drew Hildebrandt, Joseph Lichtenhan, Hamed Benghuzzi

**Affiliations:** 1 University of Mississippi Medical Center, Jackson, MS 39216, USA; dhildebrandt@umc.edu; 2 Hybrid Plastics, Hattiesburg, MS 39401, USA; lichtenhan@hybridplastics.com; 3 Department of Biology, Jackson, MS 39217, USA; j00347241@jsums.edu

**Keywords:** polyhedral oligomeric silsesquioxane (POSS), hemostat, full thickness wound, matrix metalloproteinase, tissue inhibitors of matrix metalloproteinase, wound healing

## Abstract

Wound healing is a complex dynamic biomechanical process as the body attempts to restore the integrity of traumatized or devitalized tissues. There are four stages of wound of healing that begins with hemostasis followed by inflammation, proliferation and finally weeks later wound remodeling. Full thickness wounds usually are covered with a dressing material after hemostasis, which allows for controlled hydration. We investigated the potential of a visco-liquid hemostat, polyhedral oligomeric silsesquioxane (POSS), for providing hemostasis and to maintain a microenvironment in the wound bed that would maintain moisture content and promote early re-epithelialization. We hypothesized that the hemostatic agent POSS if left in the wound bed would maintain a protective barrier and accelerate wound healing similar to using saline to irrigate the wound to keep the wound moist. We compared the early phase of wound repair (3–7 days) in a porcine full thickness wound model to evaluate the efficacy of the material. Biopsies were taken after 3 and 7 days to determine the acute response of the POSS hemostat or saline on inflammation, cell migration, concentrations of metalloproteinase (MMPs), and tissue inhibitors of metalloproteinase (TIMPs). Accelerated healing was observed in POSS treated wounds by changes in wound contraction, keratinocyte migration, and development of granulation tissue in comparison to saline treated wounds. Increased concentrations at day 3 of MMP-2, MMP-3, and in MMP-1 at day 7 in POSS treated wounds compared to saline coincide with keratinocyte migration observed in the tissue histology and changes in wound contraction. Tissue concentrations of TIMP-1 and TIMP-2 in POSS treated wounds appear to coordinate the sequence of MMP events in the healing tissue. Matrix metalloproteinase-13, a marker for tissue remodeling, was not upregulated in the early wound healing cascade in either POSS or saline treated wounds at 3 or 7 days. Overall, the data suggests POSS treatment contributed to enhanced early cell migration and wound closure compared to saline treatment.

## 1. Introduction

Wound healing is a complex dynamic biomechanical process as the body attempts to restore the integrity of traumatized or devitalized tissues. Wound healing generally can be divided into hemostatic, inflammatory, proliferative, and remodeling phases [1]. A coordinated sequence of regulated MMPs, TIMPs, and inflammatory mediators is essential to contribute to normal repair. According to the literature, the role of MMPs and TIMPs is to control the inflammatory response [2]. Dysregulation of chemical signaling in non-healing chronic wounds arrest in the inflammatory phase due to the inability of dermal and epidermal cells signal recognition [3].

It has also been proven that wounds heal quicker in a moist and clean environment. The moisture level of the environment facilitates the regulation of the milieu of tissue growth factors which coordinate epithelial cell migration within the wound bed along with wound edge contraction [4]. There are four main principles involved in wound management depending upon the wound characteristics. In wounds with excessive discharge, the exudate needs to be absorbed, while if the wound is dry, hydration is needed. Infected wounds need appropriate antimicrobial treatment, and wounds that have necrotic tissue need debridement [5].

An ideal dressing should improve hemostasis; create a moist and clean environment; prevent desiccation; remove excess exudate; be impermeable to microorganisms but allow gas exchange; be free of toxic materials; conform to the wound with minimal pain on application and removal; and be cost-effective. 

Currently available dressings work after the initial stage of hemostasis. Hybrid Plastics has developed a novel material, polyhedral oligomeric silsesquioxane (POSS), with a distinctive nanocage structure consisting of an inner inorganic framework of silicon and oxygen atoms and an outer shell of organic functional groups that can be applied to wounds to control bleeding and initiate hemostasis. In addition to supporting hemostasis, POSS is biocompatible and has unique physicochemical properties such as optical clarity and enhanced tissue adhesion. These unique properties allow visual assessment of the wound bed without the need for removal to disrupting the healing process. However, it is not currently known if the material would interfere with wound healing.

Current wound dressing materials do not meet many of the ideal characteristics, but are able to manipulate the wound environment. After irrigating the wound with saline, gauze is the most commonly used material because of cost, is readily available, and can be soaked with compounds such as iodine, petroleum or zinc and then applied. One potential problem is the gauze dressing can disrupt the wound bed upon removal, and the use of iodine and other materials contacting the wound can alter the healing rate. There are other types of dressings such as transparent flexible polyurethane sheets, which are not able to adsorb or control the exudate; similar problems occur with many of the hydrophilic dressing materials. The function is to keep the wound moist. Hydrophobic dressings include silicone, polyhexamethylene biguanide and honey dressings. Silicone dressing has been used for abnormal wound healing of keloid scars. It has been shown to soften a scar and prevent the progression of hypertrophic scar possibly by improving hydration and decreasing vapor loss [6,7]. Polyhexamethylene biguanide and honey dressings have a high which forms a mechanical barrier from the environment, long with high sugar concentrations that lead to osmotic pressure changes which makes them effective against microorganisms [8,9]. 

Overall, the process of wound healing is well described and comprises a complex series of events that appear to be coordinated through signaling proteins. Wound healing can be divided into three phases: (1) hemostasis and inflammation, (2) proliferation, and (3) maturation. Immediately following injury, a critical step is hemostasis. As the blood clots, it provides a reservoir to release and concentrate cytokines and growth factors which are necessary for cellular chemotaxis, angiogenesis, epithelialization and ultimately maturation and remodeling of the wound bed. The proliferation phase and inflammatory phase can overlap and last between 2–3 weeks following the injury [10]. In this early phase of wound healing metalloproteinases (MMPs) and tissue inhibitor of metalloproteinases (TIMPS) play a pivotal role in extracellular matrix degradation and deposition that is essential for wound re-epithelization. As the wound advances from the proliferative to the maturation phase restructuring of the unorganized extracellular matrix begins to occur. During this phase, the proteoglycans can bind to each other and fill extracellular space, lubricate the wound, and promote cell adhesion to the extracellular matrix (ECM). Decorin is a proteoglycan that participates in collagen fibril formation, and degradation of decorin can impact binding of transforming growth factor beta (TGFβ). This disruption can lead to excessive TGFβ release and improper collagen fibril formation [11]. 

Insertion of materials into the wound bed could also stimulate foreign body type reactions that could potentially alter the MMPs, TIMPs, and ECM. Insertion of hydrophobic filling materials like silicone, can trigger surface adsorption of plasma proteins forming a matrix that can initiate blood clotting, fibrinolysis, and activation of the complement system. An inflammatory reaction can occur, due to the absorption of factors on to a hydrophobic surface producing a microenvironment containing tumor necrosis factor (TNF) α, which in turn triggers maturation of extracellular matrix (ECM) proteins within fibroblasts, thereby causing fibrosis [12]. 

Polyhedral oligomeric silsesquioxane (POSS); is a hemostatic agent and can be placed in the wound to control bleeding; therefore, it is important to characterize POSS’s tissue interaction in the acute phase of wound healing. We chose to utilize a full-thickness pig skin model with damage to both the epidermis and dermis, and to follow the changes in wound size, MMPs, TIMPs, re-epithelialization, and markers of remodeling (MMP-13 and decorin) to assess the safety of using POSS as a hemostatic agent compared to using normal saline (0.9%) which is commonly used immediately after injury to irrigate the wound bed.

## 2. Materials and Methods

Test Articles: POSS and Sterile Saline: The sterile sodium chloride 0.9% (Normal saline) solution was purchased from Fisher Scientific, Pittsburgh, PA, USA (Kd Medical Inc., RGC 3290 Fisher Scientific, Pittsburgh, PA, USA). S01455-Trisilanollsooctyl Polyhedral oligomeric silsesquioxane (POSS) is commercially available and was graciously provided by Hybrid Plastics, Hattiesburg, MS, USA.

Animal Model: Under IACUC and ACURO approved protocols a series of full dermal thickness wounds were made in three, approximately 45 kg Yorkshire swine. The wounds were made on the dorsolateral surface using a custom-designed stencil that allows for standardization of the wounds to 1.5 cm × 1.5 cm, separated on each side from the next wound by 2 cm. Based upon the literature, the size of the wounds we selected usually show scabbing by 3 days with evidence of strong wound contraction between 7–9 days. This time frame allowed us to determine the efficacy of POSS as a hemostatic agent as well as if it interfered with the acute phases of wound healing. A single dose of 1.5 mL of sterile saline or POSS hemostatic gel was applied to the wound immediately after the wound creation. The wounds were photographed to assess the hemostatic capability of POSS, and again after cessation of bleeding to assess initial or baseline wound size. Additional photographs of the wounds were taken and evaluated for changes in wound size and infection at days 3 and 7, and wound biopsies were taken at 3 and 7 days to determine to assess the acute phase of wound healing. Six wounds per group per time point were compared for each treatment. Six wounds per group were used for measuring changes in wound size over time per group. Three wounds per group per time were used for biochemical evaluation of the ECM matrix. Three wounds per group per time period were used for histology. At 7 days, two of the wounds for histology per group were first evaluated using microCT, then fixed in 10% formalin for histological analysis, and one wound per group was immediately fixed in formalin for histological analysis.

Wound Bed Changes: Wound photographs including a ruler were taken at a fixed distance (37.5 cm) at 0, 3, and 7 days. For quantification, photographs of the wounds and ruler were imported into the NIH Image OS software (US National Institutes of Health, http://rsb.info.nih.gov/nih-image/) (accessed on 6 May 2024) Measures of the length and width were taken for each wound. Changes in wound area was determined at each time point by ∆A = ∆L × ∆W. The wound area for each wound then was expressed as a percent of its original size on day 0.

Protein Analysis: Tissue was collected and placed on ice, and as quickly as placed in a round bottom tube and immersed in liquid nitrogen to snap freeze. The samples were stored at −80 °C or keep on ice for immediate homogenization. For every 5 mg of tissue, 300 μL of cold extraction buffer was used. Extraction buffer consisted of 100 mM Tris (pH 7.4, 150 mM NaCl, 1 mM EGTA, 1 mM EDTA, 1% triton X-100, 0.5% sodium deoxycholate, and a Protease and Phosphatase Inhibitor Cocktail (Sigma Aldrich, St. Louis, MO, USA) at a final concentration of 1% (*v*/*v*). 

Concentration of tissue lysate protein was determined using Pierce BCA reagents and all samples were adjusted to 1 mg/mL protein. 

Biochemical Analysis: Biopsied tissues were homogenized and centrifuged and the supernatants collected and stored at −80 °C. Concentrations of Decorin, MMP-1, MMP-2, MMP-3, MMP-13, TIMP-1, and TIMP-2 were quantitated with commercially available enzyme-linked immunosorbent assays: [Matrix metalloproteinase 1 (MMP-1 Elisa Kit (porcine) MyBiosource detection range 0.312–20 ng/mL, sensitivity 0.115 ng/mL), MMP-2 Elisa Kit (porcine) (MyBiosource detection range 31.2–2000 pg/mL, sensitivity 12.7 pg/mL), MMP-3 ELISA kit (porcine) (LS Bio detection range 4.7–300 ng/mL, sensitivity 1.8 ng/mL) MMP-13 ELISA kit (porcine) (LS Bio detection range 78.13–5000 pg/mL, sensitivity 46.88 ng/mL), TIMP-1 ELISA kit (porcine) (RayBioTech detection range 18–4000 pg/mL, sensitivity 18 pg/mL), TIMP-2 ELISA kit (porcine) (RayBioTech detection range 28–7000 pg/mL, sensitivity 28 pg/mL), Decorin (porcine) (RayBioTech detection range 4.1–1000 pg/mL, sensitivity 4.1 pg/mL)]. All reaction steps were performed according to the manufacturers’ protocols.

Histology: Cross-sectional, full-thickness (4 mm) biopsies were taken from the wounds at 3 and 7 days after wounding (POSS and saline treatment) which included naïve and injured tissues. Samples were divided in half (through the center of the biopsy), fixed in 10% neutral buffered formaldehyde solution, and paraffin-embedded. Samples were sectioned and stained with hematoxylin and eosin or Masson’s trichrome. Slides were digitized using Philips slide scanner and observations were made on 5 fields of view on each slide at 400× magnification. Histologic assessments included presence of inflammatory cells, presence of POSS droplets, presence of epithelium (organized, unorganized). We used a double-blind method with two examiners (reviewer 1 was a 1st year pathology resident, and the second reviewer had fellowship training in pathology) to assess the slides. If the two examiners’ assessments were not in agreement, a third examiner (pathologist) was consulted to make a final decision.

MicroCT: A biopsy was taken to include the entire wound at 7 days. The fresh biopsy was immediately scanned in a SkyScan 1172™ microCT instrument. (Bruker, Billerica, MA, USA) Camera 10 mega pixel, Camera pixel size (μm) 11.46, image pixel size 34.40 μm, object to source 257 mm, camera to source 342 mm. Depth (bits) = 16, Exposure 790 ms, 360° rotation. Reconstruction was performed using NRecon software version 1.7.1.0.

Statistical analysis: Sigma Stat v 4.0 (Grafiti Systat Software, Palo alto, CA, USA) was used to perform statistical analysis. To compare means, Student’s *t*-test were used. The level of significance for all statistical tests was *p* < 0.05. Values in the text, tables, and figures are presented as mean ± standard deviation (SD).

## 3. Results

### 3.1. Evaluation of the Wound Beds over 7 Days

**Figure 1:**
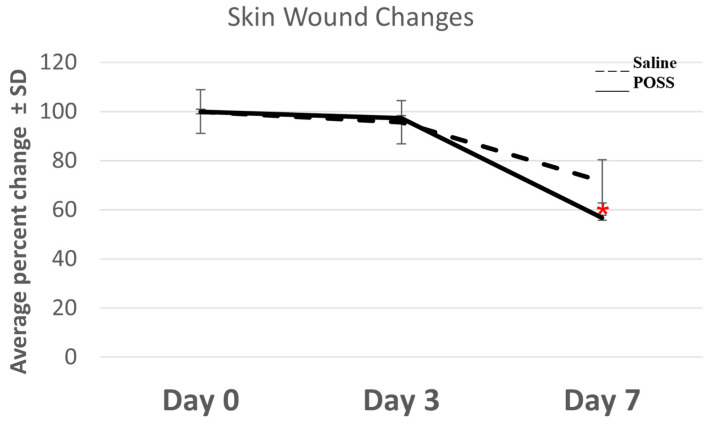
Average percent changes in wound bed area from day zero to day 7. The data are represented as mean ± SD (n = 6 wounds per group per time) * indicates statistically significant *p* < 0.05.

**Figure 2:**
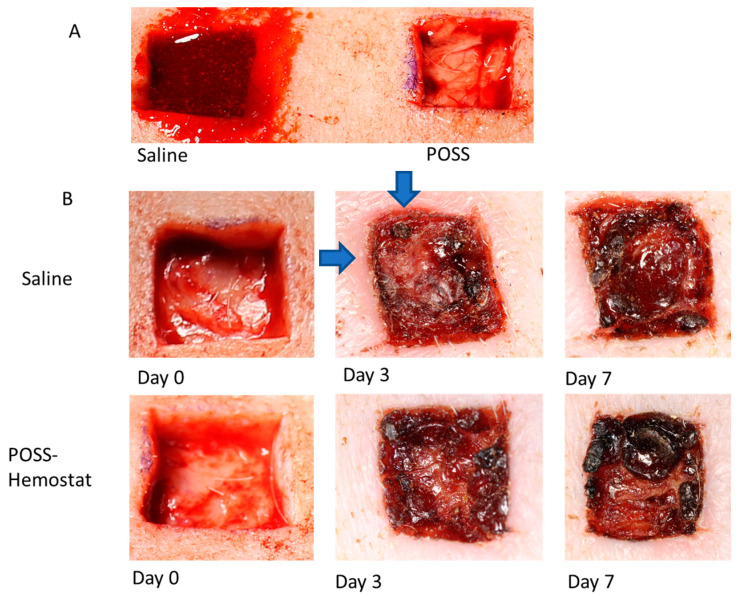
(**A**) Representative wounds on same animal one treated with saline and one treated with POSS hemostat gel minutes after wounding. (**B**) Representative photomicrographs of wounds treated with saline or POSS after cessation of bleeding at day 0, and again after 3, and 7 days. The blue arrow at day 3 in the saline treated wound indicating tissue redness around the wound bed.

On day 0, all wounds were photographed after applying saline or POSS hemostat. Measurements were taken to determine the initial wound size (Figure 1). Hemostasis was also evaluated within the first 2 min and shown in Figure 2A. Figure 2A shows a representative photograph of the initial wounds on the same animal following treatment with Saline or the POSS hemostat after 2 min. Bleeding was evident in wounds treated with saline and reduced following POSS treatment (Figure 2A). Bleeding stopped within 30 min after wounding in the saline treated wounds. Photographs of wounds and a ruler were taken after cessation of bleeding and uploaded into NIH image J, and measurements were taken to assess wound bed changes with time. No significant differences were seen in the size of the wound at day 0. However, by three days the wound size in all treated groups were smaller than at day 0 but no significant differences were detected between the groups (Figure 1 and Figure 2B). By day 7, the area of the POSS wounds was statistically smaller than wounds treated with saline alone, with visible scabs. For saline treated wounds, the percent change from day was 28.36 ± 8.46% compared to a 43.27 ± 3.67% change in the POSS treated wounds (Figure 1). In addition, redness was noticed around the wound bed at days 3 and 7 in the saline treated wounds compared to a lack of redness in the POSS treated wounds (Figure 2B).

### 3.2. Biochemical Analysis of the Wound Bed

**Figure 3:**
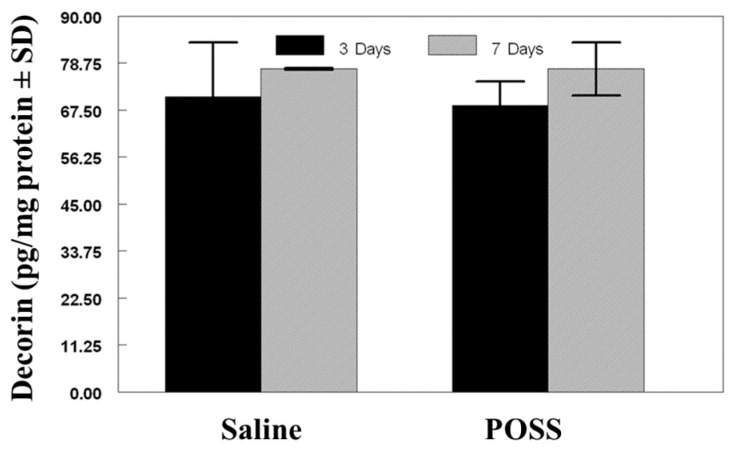
Decorin concentration (ng/mg protein) The data are represented as mean mg/protein ± SD. No statistical differences were noted for either time point.

Decorin concentration was assessed in the wounds after 3 and 7 days and ranged between 70–85 ng/mg protein (Figure 3). Differences in concentration between saline or POSS treated wounds was not found at either time point (*p* < 0.05). 

**Figure 4:**
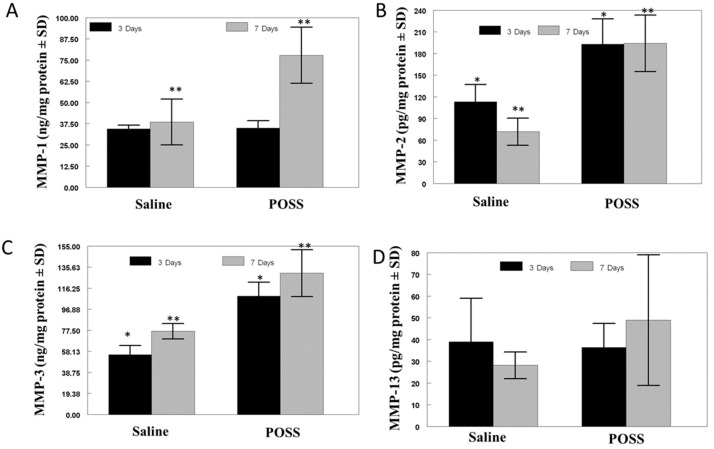
Concentrations of MMP-1 (**A**), MMP-2 (**B**), MMP-3 (**C**), and MMP-13 (**D**) in the wound bed after 3 and 7 days. (n = 3 wounds per time per treatment assay was performed in triplicate and the data are expressed as concentration mean MMP/mg protein ± SD.) (**A**) * symbol notes differences between the groups at day 3 and ** symbol notes differences at day 7. (MMP-1 at 7 days *p* < 0.001, MMP-2 and MMP-3 at * 3 and ** 7 days *p* < 0.001).

Figure 4 shows the assessment of MMP-1 (keratinocyte migration), MMP-2 (accelerated cell migration), MMP-3 (wound contraction), and MMP-13 (re-modeling). Matrix metalloproteinase-1 concentrations in the wounds were not statistically different between the groups at three days with concentrations ranging between 25–50 ng/mg protein (Figure 4A). After 7 days, the MMP-1 concentration in Saline treated wounds measured 37.5 ng/mg protein which was similar to that observed at 3 days; whereas the 7-day concentration in the POSS treated wounds averaged 76 ng/mg protein (Figure 4A). The concentrations were statistically different from the Saline treated wounds at 7 days (*p* < 0.001).

Average MMP-2 concentrations in the POSS treated wounds at both 3 and 7 days were nearly 2-fold higher than the concentration seen Saline treated wounds for both time periods (*p* < 0.001) (Figure 4B). The concentration of MMP-2 in POSS treated wounds ranged between 160–230 pg/mg protein, while the saline treated wounds had MMP-2 levels ranging between 60–130 ng/mg/protein.

A similar trend was observed in the MMP-3 concentrations where POSS treated wounds were statistically higher compared with Saline treated wounds after 3 and 7 days (*p* < 0.05) (Figure 4C).

No statistical differences were observed in MMP-13 concentrations with either time or treatment (*p* > 0.05) (Figure 4D).

**Figure 5:**
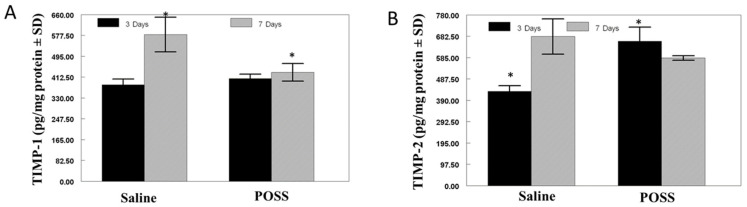
Concentrations of TIMP-1 (**A**) and TIMP-2 (**B**). The data are expressed as concentration mean TIMP/mg protein ± SD. (n = 3 wounds per time per treatment) * symbol notes differences between the groups *p* < 0.05.

TIMP-1 concentration was similar for Saline and POSS treated wounds at 3 days. By 7 days, TIMP-1 concentration increased in Saline treated wounds, while in POSS treated wounds there was no change. The increase seen in TIMP-1 at 7 days in the Saline treated wounds was statistically elevated (*p* < 0.05) (Figure 5A). In contrast, TIMP-2 was elevated in POSS treated wounds compared to saline treated wound at 3 days (*p* < 0.05) (Figure 5B). TIMP-2 concentrations increased in the Saline treated wounds which were comparable to the levels detected in wounds treated with POSS (Figure 5B).

### 3.3. Histology

**Figure 6:**
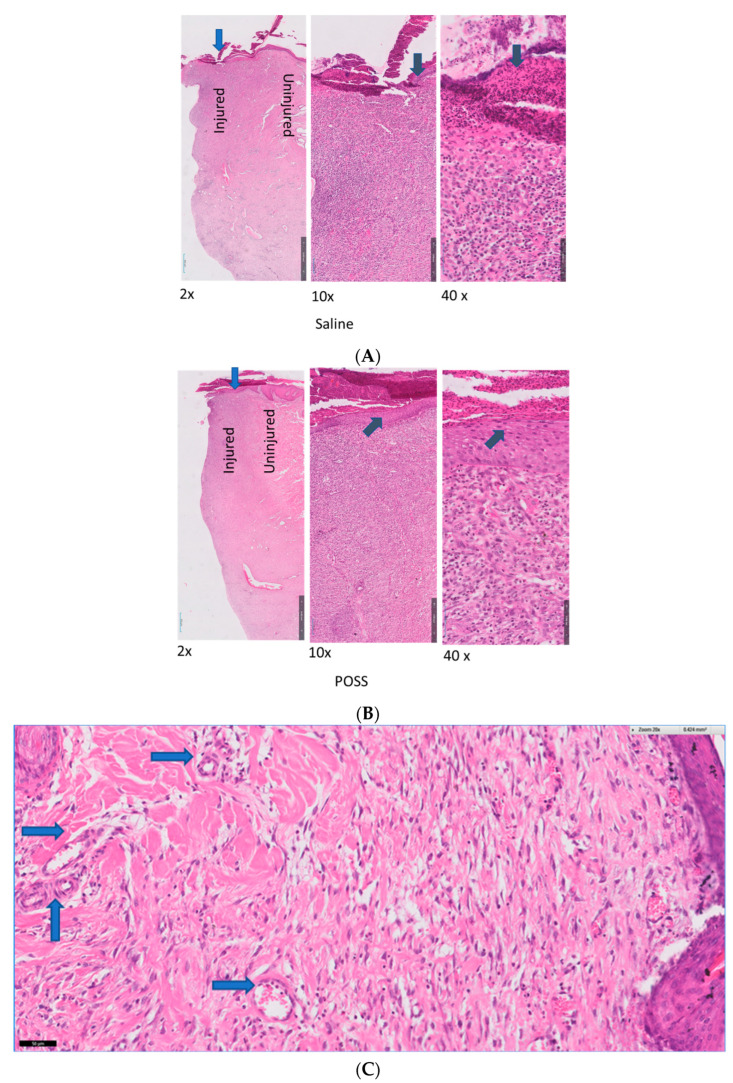
Representative tissue histology stained with hematoxylin eosin at 7 days. The blue arrow represents that location of view for 10× and 40× views and the black arrow indicates the epithelium (**A**) represents the saline treated wounds; (**B**) Represents the POSS treated wounds; (**C**) Represents areas in POSS treatment where the material showing increased vessel formation (blue arrow).

There were no histologically detectable differences in the wounds between groups after 3 days. In contrast, hematoxylin and eosin staining of the wounds showed significant histological differences between the saline (control) and POSS treatments after 7 days. In saline treated wounds we observed a large presence of inflammatory cells, limited vessel formation, evidence of cellular migration and epithelization, and disorganized collagen formation, whereas, in POSS treated wound we observed fewer inflammatory cells, new vessel formation, and enhanced re-epithelization and cellular migration. The uninjured tissue in both control saline and POSS treated groups appeared similar without evidence of an acute inflammatory response. Also, by day 7 there was a striking difference in the presence of ordered collagen fibrils and thicker epidermis (Figure 6A,B). Figure 6C shows the presence of vessel formation (blue arrows) in the POSS hemostat treated tissues.

**Figure 7:**
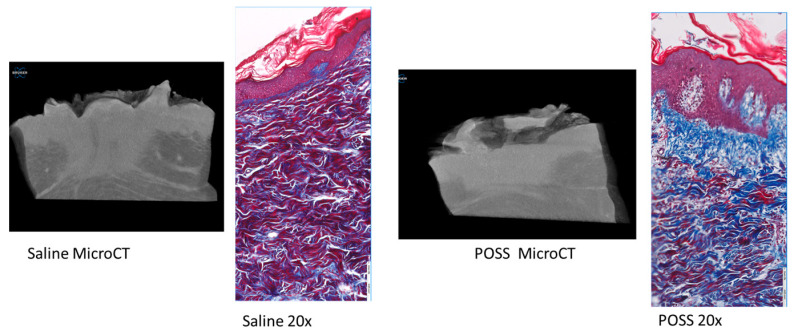
Representative photograph comparing the changes in the depth of the wound using microCT and changes in the thickness of the repairing epithelium.

**Figure 8:**
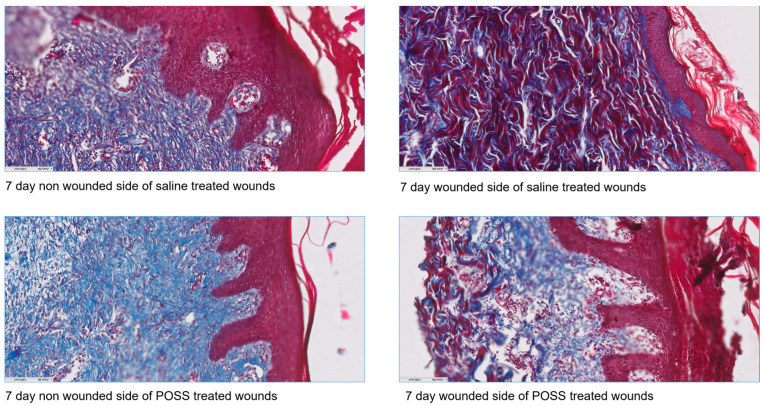
Trichrome staining of wounds showing both non-inured tissue (**left side**) and injured treated tissues (**right side**) 20× (0.425 mm^2^).

Trichrome staining of the tissue clearly shows differences in the healing tissue with less inflammation (histology) in the POSS treated wounds, along with a more intact epidermal layer (blue arrows) than in wounds treated with saline (Figure 7). Similar to what was observed using wound area analysis, micro-CT analysis of wounds at day 7 showed that saline treated wounds were deeper than POSS treated (Figure 7). Histological sections also were visualized with trichrome staining for comparison of the injured to non-injured tissue at seven days for each group (Figure 8). Striking differences can be seen in keratinization, and structures of the epidermis and dermis in the treated groups on the injured side compared to the non-injured tissue. In addition, it is evident that the POSS wounds appear to be further along in the healing process when compared with saline treated wounds.

## 4. Discussion

The wound healing cascade consists mainly of three phases, the inflammatory phase, the proliferative phase, and the maturation phase. The inflammatory phase is initiated by the formation of the clot and is followed by an increase of inflammatory cell types within the wound. This phase is short lived and followed by a longer second phase which consists of granulation, contraction and epithelialization; this then is followed by a maturation phase that consist of neutrophils, macrophages, and secreted inflammatory mediators [13]. The neutrophils secrete fibronectin which helps to initiate the migration of cells into the wound bed [14]. Stimulation of the inflammatory cells triggers release of multiple growth factors and the MMP’s and TIMPs. Release of growth factors initiate a the migration of the keratinocytes, while activity of the MMPs is coordinated by the presence of TIMPs.

In the acute phase, hydrophobicity of POSS can trigger adsorption of plasma proteins onto the surface of POSS forming a protein matrix which ultimately triggers the immune system, inducing hemostasis. However, it has not been shown if interfering with absorption of protein onto the surface of POSS would alter the acute wound healing cascade.

Tissue concentrations of MMP-1 and MMP-3 had similar trends at 3 and 7 days following wounding. MMP-1 is expressed by keratinocytes is thought to occur about a week after injury, and regulates the interaction of keratinocyte with type I collagen in the re-epithelial phase [15]. The increase in MMP-1 one week after injury suggest active re-epithelialization of the wound which is also consistent with the wound histology at 7 days. MMP-3 is expressed in fibroblasts and by the proliferating keratinocytes during wound healing [16,17], is responsible for activation of cytokines and growth factors and MMP-1 [17,18]. It is thought the role of MMP-3 is to enhance reannealing of the wound edge. It appears to play a major role in cell migration and proliferation during the repair process wound edge by promoting cell migration and proliferation in the wound during the repair process. We found that MMP-3 was activated early on in POSS treated wounds and remained elevated at 7 days; in contrast, saline treated wounds exhibited only a modest increase from day 3 to day 7.

The gelatinase MMP-2, mediates platelet adhesion and aggregation [19]. A single treatment with POSS increased MMP-2, which supports its use as a hemostatic agent. Gelatinases also are critical in the early stages of wound repair to dislodge the attachment of bacterial biofilms. Additional studies have shown that MMP-2 also coincides with keratinocyte migration and healing [20,21,22]. Therefore, the early increase in MMP-2 may be attributed to platelet mediated events while the late rise at 7 days along with MMP-1 and MMP-3 suggest enhanced migration of the keratinocytes.

MMP-13 is considered an interstitial collagenase, is expressed by fibroblasts in chronic wounds and plays a role in maturation of granulation tissue [23,24,25]. It is probable that we did not see differences in MMP-13 concentrations between wounds treated with POSS or saline because those wounds were in the early phase of healing, before MMP-13 would be expected to be expressed TIMP-1 is abundantly expressed by epithelial cells, spindle-shaped, fibroblast-like, macrophage-like stromal cells, as well as by endothelial cells and can inhibit MMP-1, MMP-2, and MMP-13 [26,27]. In histological studies of porcine wounds, TIMP-1 was detected until re-epithelialization, most notably in the basal keratinocytes [28,29]. TIMP-1 also is crucial for limiting leukocyte extravasation and vascular permeability [30]. The early rise in TIMP-1 in the POSS treated wounds may have played a role in limiting inflammatory cell types; this clearly is evident by the red and swollen gross appearance of the wounds as well as histological data at days 3 and 7 showing fewer inflammatory filtrates in the POSS treated wounds. In normally healing wounds, TIMP-2 protein localizes under the migrating epithelial cells and has been shown to inhibit MMP-1, MMP-2, and MMP-3. Although TIMP-2 has been shown to accelerate cell migration, which should promote wound healing, it also has been shown to exhibit uncontrolled activity in chronic, ulcer-type wounds, which would impair wound healing. Because of the short duration of the current study, we cannot postulate a role for TIMP-2 in the healing that we observed.

Decorin has been shown to influence tissue tensile strength and cellular phenotypes and is involved with extracellular matrix assembly [31,32]; however, the exact mechanism of action for decorin in wound healing is not clear. In clinical practice, decorin has been used in conjunction with different skin substitutes and additional ECM matrix proteins matrix proteins; this then leads to successful healing of chronic wounds by regulating the activity of a number of growth factors and preventing excessive fibrosis tissue response [33,34]. The lack of difference in decorin in the early phase of wound healing after exposure to POSS is not surprising since like MMP-13 is considered to be more important during the remodeling phase.

Polyhedral oligomeric silsesquioxane, like silicone, is stable and non-toxic which makes it an ideal medical material. Once the hydrophobic materials are placed into the tissue, microdroplets of various sizes can from. Microdroplets ranging in size from 20–100 µm^2^ can flow along tissue planes based upon gravity. These larger droplets cannot be engulfed by the cell and instead activate the cells on the surface of the material initiating a foreign body reaction that over time can create a fibrous capsule formation on the smooth surface of the POSS droplets. In this study the minimal presence of foreign body cells along with the minimal presence of inflammatory cells by 7 days suggests the material can be used as a hemostatic agent in full thickness wounds. We did not evaluate lymph nodes to see migration of the material in this study, but the size of the POSS material placed into wound bed was well over 200 µm^2^ and would not be carried by macrophage cells in the lymphatic tissues. Plus, the use of silicone oil ranging between 1000–5000 centistoke has been used for retinal detachment repair since 1962 [35] and more recently off label for dermal fillers [36]. The lack of movement of the material after placement suggest that it is not carried in lymphatics or migrates away from the area of injection. In addition, The POSS material used in this study (POSS SO1455) is 26,289 centistokes at 37 °C which is equivalent to 27,500 cpoise which is equivalent to 27.5 Pa s and has limited flow properties that would suggest movement into the tissues.

Wound healing requires a desirable microenvironment, in which moisture is on the most essential factors [4,37,38]. The hydrophobic nature of POSS serves as a barrier to for vapor loss and protects the moister content of the tissue. The ability of POSS to maintain a suitable tissue microenvironment conducive for MMPs and TIMPs to interact is crucial for normal wound healing. For example, wounds with a low moisture microenvironment can hinder migration of keratinocyte cell types while promoting fibroblast cell types to proliferate in the wound bed creating abnormal scar tissue. Protection of moisture content allows for faster re-epithelialization that appears similar to naïve non injured tissues. Overall, POSS appears to be beneficial as a wound coverage option in controlling the hemostasis, inflammatory, and proliferative phases of wound healing.

## 5. Conclusions

Early increases in MMP-3 at 3 day coincides with the changes in wound contraction seen in the POSS treated wounds and suggests a decrease in the healing time. This is also demonstrated by the early increase in MMP-2 at day 3 which suggests increased cellular migration into the wound bed, and at 7 days the increase in MMP-1 is indicative of keratinocyte migration which is reflective of the histology. MMP-13, a marker for remodeling and not upregulated in the early wound healing cascade. TIMP-1 and TIMP-2 most likely coordinate the sequence of events in the healing tissues. The biochemistry and the histology closely follow the overall migration of the keratinocytes and wound closure. Overall, POSS technology can be used as a potential dermal protectant that can be applied to an open wound to preserve the integrity of the tissue, maintain the moisture content, and increase wound healing.

## Data Availability

The original contributions presented in the study are included in the article.
